# Reactions to environmental allergens in cats with feline lower airway disease

**DOI:** 10.3389/fvets.2023.1267496

**Published:** 2023-12-07

**Authors:** Birte F. Hartung, Ralf S. Mueller, Jana Gauss, Tamara Weitzer, Teresa M. S. A. Boehm, Jelena Palić, Bianka Schulz

**Affiliations:** ^1^LMU Small Animal Clinic, Ludwig Maximilian University of Munich, Munich, Germany; ^2^Statistical Consulting Unit StaBLab, Department of Statistics, Ludwig Maximilian University of Munich, Munich, Germany; ^3^Vet Med Labor GmbH Division of IDEXX Laboratories, Kornwestheim, Germany

**Keywords:** immunoglobulin E, intradermal test, feline asthma, chronic bronchitis, feline atopic syndrome

## Abstract

**Objectives:**

Aeroallergens have been discussed as potential triggers for feline asthma (FA), which can be induced experimentally by allergen sensitization. To date, only few studies have investigated reactions to environmental allergens in cats with naturally occurring feline lower airway disease (FLAD). The aim of the study was to compare results of intradermal testing (IDT) and serum allergen-specific immunoglobulin E-(IgE) testing (SAT) in cats with FLAD, and to investigate possible associations with allergen exposure.

**Material and methods:**

Eight cats with eosinophilic airway inflammation (EI), ten cats with mixed inflammation (MI), six with neutrophilic inflammation (NI), and 24 healthy cats (HC) were included. Cats diagnosed with FLAD were assigned to the different inflammatory groups based on bronchoalveolar lavage fluid (BLAF) cytology. SAT was performed in all cats; IDT was only carried out in cats with FLAD. Information about the cats' environment and potential allergen exposure was obtained using an owner questionnaire.

**Results:**

In comparison to 83% of HC with positive reactions on SAT only 52% of cats with FLAD had positive responses (*p* = 0.051). Significantly more positive reactions per cat were detected on IDT than on SAT (*p* = 0.001). No significant difference was found for positive reactions per cat on SAT when compared between HC, NI, EI, and MI (*p* = 0.377). Only “slight” agreement was found for most allergens when reactions obtained in both tests in cats with FLAD were compared, except for “moderate” agreement for English plantain (k = 0.504) and *Alternaria alternata* (k = 0.488). Overall, no clear association between the cats' environment and allergen reactions were detected.

**Conclusions and clinical importance:**

Interpretation of allergy test results in cats with FLAD should be done in the context of clinical signs and individual factors.

## 1 Introduction

Feline lower airway disease (FLAD) is a common inflammatory condition in cats. Recent studies support the idea that feline asthma (FA) and feline chronic bronchitis (CB) are the two most common inflammatory diseases falling under this term (1–4). However, a clear discrimination between these two phenotypically similar syndromes and their potential etiologies could not be established (5, 6). That raises the question whether FA and CB are two different disease entities or if they are just arising from the same underlying cause with variations in their inflammatory profile (6, 7). To date, the only attempt to differentiate between FA and CB is based on cell differentiation of bronchoalveolar lavage fluid (BALF) cytology, suggesting an eosinophilic inflammation (EI) representing FA, and a sterile neutrophilic inflammation (NI) typical for CB (2, 8). Recently, a subdivision of so-called “mixed inflammation” (MI) has been introduced for further categorization (9–13). It is assumed that MI is a consequence of chronic EI, resulting in damage of airway epithelium, and therefore leading to immigration of neutrophils (1, 5, 14). According to recent definitions, FA is assigned to the feline atopic syndrome comprising allergic diseases of the skin, respiratory and gastrointestinal tract in cats (15).

In multiple studies FA could be induced experimentally via parenteral sensitization and aerosol challenge using common environmental allergens. Therefore, it is highly suggestive that an allergic reaction is an underlying major cause for the inflammation seen with FA (16–20). Currently, standard therapy consists of inhaled or systemically administered glucocorticoids, often in combination with bronchodilators (1, 8, 14, 21). Even though it could be demonstrated that clinical signs improve significantly with this therapy (21, 22), subclinical inflammation may continue to damage the lower airways in some cats (23), and side effects associated with long-term use of corticosteroids can pose a risk for patients (24, 25). Therefore, it has been suggested to utilize allergen immunotherapy (AIT) as a causative treatment of a possible allergic etiology, which has been successfully performed in experimental studies using FA models (17, 26, 27). For successful AIT, it is essential to identify patient-relevant allergens which can be supported by serum or skin allergy tests (20). In one study using an experimental model of cats sensitized to Bermuda grass allergen or house dust mite allergen (HDMA), IDT revealed a greater sensitivity compared to serum allergen-specific immunoglobulin E-(IgE) testing (SAT), although SAT showed higher specificity (18). In naturally occurring FA, IDT and SAT results have been compared in a pilot study which showed significantly more individual positive reactions on IDT and SAT in cats with FLAD compared to healthy cats (HC) (28). Recent studies identified responses to house dust mites and storage mites (SM) as common reactions on SAT in cats with FLAD; however, IDT was not performed in these investigations and environmental factors were not specifically assessed (9, 29).

Therefore, the aim of the study was to prospectively investigate agreement between IDT and SAT results in cats with FLAD and to assess possible correlations with environmental factors.

## 2 Materials and methods

The prospective case-controlled study was approved by the Ethics Committee of the Centre for Clinical Veterinary Medicine of the Ludwig Maximilian University of Munich, Germany (No. 239-16-11-2020) and was conducted between December 2020 and June 2022.

### 2.1 Study population

All cats with FLAD were privately owned patients of the Small Animal Clinic of the Ludwig Maximilian University of Munich, Germany, presenting with respiratory complaints. HC were presented for health care check-up, vaccination titer check or dental care. Inclusion criteria were clinical signs indicative of FLAD (chronic cough, wheezing, episodes of tachypnea or respiratory distress), radiographic evidence of bronchial or bronchointerstitial lung pattern, elevated total cell count in BALF >400 cells/μl (30, 31), and cytological evidence of sterile airway inflammation. For BALF cytology, 200 cells of a cytospin sample were assessed by the same board-certified clinical pathologist. Inflammation was categorized as eosinophilic (eosinophils >20% with neutrophils <14%, or eosinophils >50%), mixed (eosinophils 20%-50% and neutrophils >14%), or neutrophilic (neutrophils >14% and eosinophils <20%) inflammation as previously described (6). In addition, aerobic bacterial cultures and *Mycoplasma* spp.-PCR were performed on all BALF-samples. Cats with positive *Mycoplasma spp*.-PCR were not excluded, as *Mycoplasma* spp. has been described as a commensal organism in the lower airways of cats (32). Additionally, cats with a positive *Mycoplasma* spp. PCR received oral Doxycycline for 3 weeks, yet symptoms persistet. Deworming status of the cats included in this study could not be determined. In all outdoor cats, Baermann fecal analysis was performed to rule out lungworm disease. Exclusion criteria were the administration of glucocorticoids within 4 weeks or of antihistamines within 2 weeks prior to examination, detection of respiratory disease other than FLAD, presence of severe systemic disease, or unstable patients exhibiting respiratory distress at presentation. Inclusion criteria for HC were an unremarkable clinical examination and the absence of a history of respiratory or dermatological diseases.

### 2.2 Sample collection

#### 2.2.1 Diagnostic workup

All owners of cats with FLAD and HC filled out a questionnaire on environmental factors of their cats (Supplementary material). In cats suspicious for FLAD, a thorough clinical examination and thoracic radiographs (ventrodorsal and laterolateral right-sided) were carried out. Blood samples were collected for a complete blood cell count and serum biochemistry analysis. In cats with outdoor access, Baerman analysis from a three-day fecal collection was carried out to exclude lung worm infection. As *Dirofilaria immitis* is not endemic in Germany, and none of the patients originated from or had traveled to an endemic area, heart worm testing was not performed. Prior to anesthesia, cats received 0.01 mg/kg terbutaline subcutaneously (Bricanyl^®^, AstraZeneca, Wedel, Germany) to minimize the risk of bronchoconstriction, and 0.2mg/kg Butorphanol (Butorgesic^®^, cp pharma, Burgdorf, Germany) was given intravenously for mild sedation. Anesthesia was induced and maintained intravenously with Alfaxan (Alfaxalon^®^, Jurox, Rutherford, Australia). Blind bronchoalveolar lavage was performed in cats with FLAD according to a previously published protocol (32). Each BALF was submitted for aerobic bacterial culture (Institute for Infectious Diseases and Zoonoses of the Ludwig Maximilian University of Munich, Germany) and *Mycoplasma-*spp.-PCR (Synlab Laboratory, Augsburg, Germany). Total cell count of BALF was determined directly after sample collection, and stained native and cytocentrifuged BALF-smears were evaluated cytologically by a board-certified clinical pathologist (JP). For this purpose, two direct smears and two cytospin preparations were stained with modified Wright's stain. Multiple microscopic fields were examined to obtain a total of 200 cell differential count.

#### 2.2.2 Intradermal testing

During anesthesia for the diagnostic workup, IDT was performed according to a published protocol (33). For this procedure the lateral aspect of the left thorax was carefully clipped, and injection sites were marked with a water-soluble pen. In total, 39 injections were administered intradermally with a volume of 0.08 ml each, including a negative (saline) and a positive control (histamine phosphate) (Table 1). After 15 and 25 min, injection sites were evaluated for diameter of the wheal, turgidity, and erythema in comparison to positive and negative control. The local reactions were graded from 0 (negative control) to 4 (positive control) and results were recorded. Evaluation was carried out by a board-certified dermatologist as well as dermatology residents, who were familiar and experienced with the procedure.

**Table 1:** Allergens, number of reactions to allergens in cats with FLAD and HC and agreement between tests.

**IDT**		**SAT**			**Agreement between IDT and SAT (κ-value)**
**Allergens tested in both tests**		**Allergens tested in both tests**			
**Allergen**	**FLAD (Number of positive reactions)**	**Allergen**	**FLAD (Number of positive reactions)**	**HC (Number of positive reactions)**	**(kappa could only be calculated when there was at least one positive reaction in each test)**
Rapeseed	5	Rapeseed	2	1	0.145
Goosefoot	4	Goosefoot	2	5	0.129
Ragweed	8	Ragweed	0	2	IDT 34% positive results, 0% positive results on SAT
Sheep sorrel	6	Sheep sorrel	0	3	In IDT 24% positive results, 0% positive results on SAT
Mugwort	4	Mugwort	0	1	IDT 25% positive results, 0% positive results on SAT
English plantain	5	English plantain	2	2	0.504
Orchard grass	2	Orchard grass	0	0	IDT 5% positive results, 0% positive results on SAT
Timothy grass	7	Timothy grass	5	4	0.034
Perennial ryegrass	4	Perennial ryegrass	0	2	IDT 15% positive results, 0% positive results on SAT
Bermuda grass	9	Bermuda grass	0	2	IDT 43% positive results, 0% positive results on SAT
Kentucky bluegrass	1	Kentucky bluegrass	5	5	IDT 0% positive result, on SAT 24% positive results
Mold mite *(Tyrophagus putrescentiae)*	3	Mold mite *(Tyrophagus putrescentiae)*	1	6	0.068
American house dust mite *(Dermatophagoides farinae)*	5	American house dust mite *(Dermatophagoides farinae)*	7	12	0.080
Grocers‘itch mite *(Lepidoglyphus destructor)*	6	Grocers‘itch mite *(Lepidoglyphus destructor)*	0	0	IDT 29% positive results, 0% positive results on SAT
European house dust mite	5	European house dust mite	0	0	IDT 15% positive results, 0% positive results on SAT
Flour mite *(Acarus siro)*	5	Flour mite *(Acarus siro)*	0	4	IDT 24% positive results, 0% positive results on SAT
Flea	3	Flea	1	1	0.050
*Alternaria alternata*	4	*Alternaria alternata*	3	8	0.488
*Aspergillus fumigatus*	2	*Aspergillus fumigatus*	2	6	0.105
*Cladosporium herbarum*	3	*Cladosporium herbarum*	2	5	0.167
*Malassezia*	7	*Malassezia*	0	2	IDT 34% positive results, 0% positive result on SAT
Upright pellitory	5	Stinging nettle	1	1	
Creeping bentgrass	2	Dandelion	1	2	
Perennial grass	10	Oat	0	2	
Cough grass	4	Rye	0	2	
Goldenrod	6	Birch	2	1	
Beech	1	Olive tree	3	3	
Hazel	6	Plane tree	3	2	
Silver poplar	5	Willow	6	9	
Cockroach	0	Pine	2	1	
House fly	2	Privet	4	5	
Mosquito	4	Cypress	1	1	
Culex	5	Oak	0	1	
Biting midges	5	American elm	0	1	
Horse fly	10				
Sheep's wool	3				
Goose feather	2				

FLAD, feline lower airway disease; HC, healthy cats.

#### 2.2.3 Serum allergen-specific immunoglobulin E-(IgE) testing

In HC, left over material was used for analysis. Serum had been collected for general check-up, vaccination titer check or blood work collected prior to anesthesia for dental care. All serum samples were stored at −80°C immediately after centrifugation. The samples were then sent in groups to the laboratory (Nextmune S.L.U., Madrid, Spain) for specific IgE-antibody detection for 34 different allergens (Table 1). On the way to the external laboratory, one batch of serum samples was lost in mail transit, which reduced the total number of SATs carried out to 21 instead of 24 in the group of cats with FLAD. All sera were tested against cross-reactive carbohydrate determinants (CCDs) using indirect ELISA (CCD-screening). Sera then were diluted 1/6 in buffer solution containing 1% bovine serum albumin and 0.1% Tween 20. Samples with positive specific IgE against CCDs were processed after adding a CCD-blocker before dilution. For blocking, serum was incubated for 1 h at room temperature with a CCD-blocker. After that, samples were added to 96-well plates coated with allergens in duplicates. The plates were incubated at 4 °C overnight. Next, plates were washed four times with washing buffer, and monoclonal antibody was added, followed by incubation at 4 °C for 2 h. Thereafter, plates were washed six times with washing buffer, and p-nitrophenyl phosphate substrate (Moss, MD, USA) was added to the wells. After 30 min incubation time at room temperature, 1 N sodium hydroxide (NaOH) was added to stop the reaction. Absorbances were read at 405 nm using a spectrophotometer. Positivity was defined according to a protocol established by Nextmune expressing results in ELISA absorbance units.

### 2.3 Statistical analysis

All analyses were performed using commercially available software (IBM^®^, SPSS Statistics version 28.0.1.0). Nonparametric tests were used, as data was not normally distributed. Mann-Whitney U test was used for age comparison between cats with FLAD and HC. In addition, it was used to compare positive reactions per cat in IDT and SAT with allergens appearing in both tests (*n* = 21) as well as positive reactions in indoor- and outdoor cats. Comparison of more than two groups was performed with Kruskal–Wallis test, which was applied for positive reactions on SAT and IDT between the different groups, and for positive reactions between specific allergen groups. When significance was demonstrated, a *post-hoc* Bonferroni correction was performed. Fisher's-exact test was applied for evaluation of an association of SM reactions and dry food diet. In addition, it was used to compare the number of cats with at least one reaction on SAT between groups. Agreement of IDT and SAT results for allergens appearing in both tests was evaluated using Cohen's *kappa*. “Poor” to “almost perfect” agreement was reported as previously defined (Table 2) (34). Eleven allergens were compared only descriptively as *kappa* was by definition zero given the fact that one test showed no positive reactions for a specific allergen. Seasonality was assessed descriptively. For all tests *P* < 0.05 was considered significant.

**Table 2:** Standard interpretations of Cohen's Kappa (34).

**Kappa statistic**	**Strength of agreement**
<0	Poor
0.01–0.20	Slight
0.21–0.40	Fair
0.41–0.60	Moderate
0.61–0.80	Substantial
0.81–1.00	Almost perfect

## 3 Results

Twenty-four client-owned cats diagnosed with FLAD and 24 HC who served as a control group were included. Four cats suspected of having FLAD were previously excluded due to physiological BALF cytology. Of the remaining 24 cats with FLAD, eight showed EI, ten MI, and six NI on BALF cytology (Table 3). Four cats had positive PCR results for *Mycoplasma spp*. and two cats had low levels of bacterial growth after enrichment on BALF culture: growth of *Pasteurella multocida, Neisseria zoodegmatis*, and *Staphylococcus felis* in one cat, and *Pasteurella* spp. in another patient (Table 3).

**Table 3:** Signalement, clinical, laboratory, and microbiological parameters in cats with FLAD.

**Cat no**.	**Age year**	**Breed**	**BALF inflammatory type**	Blood Eosinophils^*^(10^9^/*l*)	**BALF bacterial culture**	**BALF total cell count (cells/μL)**	***Mycoplasma spp*. PCR**	BALF Eosinophils^**^(%)	BALF Neutrophils^**^(%)	BALF Macrophages^**^(%)	**Seasonality**
1	2	Siamese	EI	0.61	Neg	1,250	Neg	70	8	22	None
2	2	DSH	EI	0.43	Neg	1,330	Neg	26	6	68	None
3	4	DSH	EI	0.53	Neg	3,260	Neg	43	12	45	IU
4	1	Oriental Shorthair	EI	0.49	Neg	1,140	Neg	26	8	66	None
5	9	Siamese	EI	0.23	Neg	870	Neg	35	2	63	Spring
6	3	Siamese	EI	**1.28**	Neg	1,740	Neg	85	6	9	None
7	2	Norweigan Forest	EI	**0.75**	Neg	2,060	Neg	62	7	31	None
8	7	DSH	EI	0.29	Neg	2,030	Neg	38	14	48	None
9	7	Russian Blue	MI	**0.76**	Neg	830	Pos	27	47	26	IU
10	11	DSH	MI	**0.89**	Neg	2,470	Neg	22	66	12	None
11	15	DSH	MI	**0.74**	Neg	2,270	Pos	21	68	11	None
12	10	DSH	MI	0.36	Neg	410	Neg	33	53	14	None
13	9	DSH	MI	**0.63**	Neg	2,710	Neg	39	42	19	Spring
14	3	DSH	MI	**2.35**	Neg	2,210	Neg	32	47	21	Summer
15	11	DSH	MI	0.52	Neg	2,310	Neg	27	64	9	None
16	2	Maine Coon	MI	0.5	Neg	1,030	Neg	41	21	36	None
17	1	Maine Coon	MI	**1.23**	Neg	910	Neg	25	18	57	None
18	2	Maine Coon	MI	0.45	*P. multocida Neisseria zoodegmatis Staphylococcus felis* (AE)	4,950	Neg	20	24	56	None
19	3	DSH	NI	0.35	Neg	11,350	Pos	1	95	8	None
20	11	Siamese	NI	0.5	Neg	2,060	Neg	2	72	26	None
21	11	Norwegian Forest	NI	0.33	Neg	2,560	Pos	2	91	7	None
22	13	DSH	NI	**2.38**	Neg	3,630	Neg	2	82	14	Summer
23	3	DSH	NI	**0.69**	*Pasteurella spp* (AE)	850	Neg	7	21	70	Summer
24	4	DSH	NI	0.23	Neg	3,620	Neg	11	21	68	None

EI, eosinophilic inflammation; MI, mixed inflammation; NI, neutrophilic inflammation; AE, bacterial growth after enrichment; IU, information unavailable. ^*^Values above reference range marked in bold numbers. ^**^Cell distribution in BALF-cytology in %. Cats 23 and 13 had dermatitis and puritus issues. Cat 23 had pruritus only in summer and Cat 13 had puritus the whole year.

Of the 24 cats with FLAD ten were female spayed (42%), and 14 male neutered (58%). HC consisted of ten female spayed (42%) and one intact female (4%), and twelve male neutered (50%), and one intact male (4%). Breeds of FLAD cats included Domestic Shorthair (*n* = 13), Siamese (*n* = 4), Maine Coon (*n* = 3), Norwegian Forest (*n* = 2), Russian Blue (*n* = 1), and Oriental Shorthair (*n* = 1) (Table 3). Breeds of HC were Domestic Shorthair (*n* = 20), Siberian (*n* = 1), Russian Blue (*n* = 1), Norwegian Forest (*n* = 1) and British Shorthair (*n* = 1). Median age did not differ significantly between cats with EI (2.5, range: 1–9 years), MI (8.0, range: 1–15 years), NI (7.5, range: 3–13 years) and HC (3.0, range: 1–13 years) (*p* = 0.118). In both groups most cats were kept indoors (63% with FLAD and 83% HC) and lived in an urban environment (79% FLAD and 88% HC). Two cats with FLAD showed additional signs of dermatitis and pruritus, one of them with more severe dermatological and respiratory signs during summer (Table 3). The duration of time since first onset of clinical signs ranged from 3 months to 6 years (median 1.5 years), with cough *n* = 22 (92%) being the most predominant clinical sign besides tachypnea *n* = 3 (13%), episodes of respiratory distress *n* = 4 (17%) and wheezing *n* = 2 (8%). Blood eosinophilia was present in ten cats with FLAD (42%) (Table 3).

More HC 20/24 (83%) showed at least one positive allergen reaction on SAT compared to cats with FLAD 11/21 (52%) (*p* = 0.051). On IDT 22/24 (91%) cats with FLAD showed positive allergen reactions. When results of IDT and SAT for cats with FLAD were compared, significantly more positive reactions per cat were found on IDT (*p* = 0.001). No significant difference could be detected for median positive reactions per cat on SAT between cats with FLAD and HC (*p* = 0.185), nor when compared between NI, EI, MI, and HC (*p* = 0.377) (Tables 4A, B).

**Table 4A:** Positive reactions per cat on IDT (*n* = 37) in cats with FLAD.

**EI (*n =* 8)**		**MI (*n =* 10)**		**NI (*n =* 6)**		***p-*value**	**FLAD (*n =* 24)**	
**median**	**range**	**median**	**range**	**median**	**range**		**median**	**range**
8.5	5–14	2.5	0–12	5.0	5–10	0.008^*^	5.0	0–14

^*^Post-hoc Bonferroni correction: EI has significantly more positive reactions compared to MI (p = 0.002), but not when compared to NI (p = 0.195).

**Table 4B:** Positive reactions per cat on SAT (*n* = 34) in cats with FLAD and HC.

**EI (*n =* 7)**		**MI (*n =* 9)**		**NI (*n =* 5)**		**HC (*n =* 24)**		***p*-value**	**FLAD (*n =* 21)**	
**median**	**range**	**median**	**range**	**median**	**range**	**median**	**range**		**median**	**range**
5.0	0–9	1.0	0–6	0.0	0–10	2.0	0–29	0.377	1.0	0–10

IDT, intradermal test; SAT, serum allergen-specific immunoglobulin E test; EI, eosinophilic inflammation; MI, mixed inflammation; NI, neutrophilic inflammation; HC, healthy cats; FLAD, feline lower airway disease.

When agreement between IDT and SAT results was assessed for the ten allergens evaluated in both tests, “moderate agreement” was observed for the allergens *Alternaria alternata* (κ = 0.488*)* and English plantain (κ = 0.504). For the other eight allergens, agreement was only “slight” (Table 1).

On SAT the most common allergen reaction in cats with FLAD (*n* = 7) and HC (*n* = 12) was American house dust mite (*Dermatophagoides farinae*), followed by fungi in HC with *Alternaria alternata* (*n* = 8) and *Aspergillus fumigatus* (*n* = 6) (Table 1).

When median positive reactions per cat were compared for the three inflammatory types regarding different allergen groups on IDT, reactions to tree pollen were significantly more common in cats with EI than in those with NI and MI. In addition, cats with EI and those with NI reacted significantly more commonly against allergens of the insect group than cats with MI (Table 5A). There were no significant differences for the comparison of median positive reactions per cat in allergen groups tested on SAT (Table 5B). Total positive reactions for all groups of cats are listed in Tables 5A, B.

**Table 5A:** Total positive reactions in cats (and median positive reactions per cat) with different type of inflammation on IDT, reactions to groups of allergens.

**IDT (35 allergens)**	**EI (*n =* 8)**		**MI (*n =* 10)**		**NI (*n =* 6)**		***p*-value**
	**Total positive reactions**	**Median positve reactions (range)**	**Total positive reactions**	**Median positve reactions (range)**	**Total positive reactions**	**Median positve reactions (range)**	
Grasses (*n =* 9)	17	2.00 (0–5)	13	1.00 (0–3)	15	2.50 (1–4)	p = 0.285
Weeds (*n =* 7)	15	2.00 (0–4)	13	1.00 (0–5)	9	1.50 (0–2)	p = 0.416
Trees (*n =* 3)	11	2.00 (0–3)	1	0.00 (0–1)	0	0.00 (0–0)	p = 0.007^*^
Fungal (*n =* 4)	9	1.00 (0–3)	4	0.00 (0–2)	2	0.00 (0–1)	p = 0.353
Mites (*n =* 5)	11	1.00 (0–3)	6	0.50 (0–2)	7	1.00 (0–3)	p = 0.229
Insects (*n =* 7)	14	1.50 (1–4)	5	0.50 (0–1)	10	2.00 (0–4)	p = 0.021^**^

^*^Post-hoc Bonferroni correction: EI significantly more positive reactions than NI (p = 0.007) and MI (p = 0.007). No significant difference between NI and MI (p = 0.731). ^**^Post-hoc Bonferroni correction: EI significantly more positive reactions than MI (p = 0.010). NI significantly more positive reactions than MI (p = 0.044). No significant difference between EI and NI (p = 0.731).

**Table 5B:** Total positive reactions in cats (and median positive reactions per cat) with different type of inflammation on SAT, reactions to groups of allergens.

**SAT (34 allergens)**	**EI (*n =* 7)**		**MI (*n =* 9)**		**NI (*n =* 5)**		**HC (*n =* 24)**		***p*-value**
	**Total positive reactions**	**Median positve reactions (range)**	**Total positive reactions**	**Median positve reactions (range)**	**Total positive reactions**	**Median positve reactions (range)**	**Total positive reactions**	**Median positve reactions (range)**	
Grasses (*n =* 7)	5	0.00 (0–2)	3	0.00 (0–1)	2	0.00 (0–3)	17	0.00 (0–6)	p = 0.733
Weeds (*n =* 8)	4	0.00 (0–3)	1	0.00 (0–1)	3	0.00 (0–2)	17	0.00 (0–6)	p = 0.838
Trees (*n =* 9)	9	0–00 (0–4)	7	0.00 (0–4)	5	0.00 (0–4)	24	1.00 (0–8)	p = 0.808
Fungal (*n =* 4)	4	0.00 (0–3)	3	0.00 (0–3)	0	0.00 (0–0)	21	0.00 (0–4)	p = 0.124
Mites (*n =* 5)	5	1.00 (0–2)	2	0.00 (0–1)	1	0.00 (0–1)	22	1.00 (0–3)	p = 0.114
Insects (*n =* 1)	1	0.00 (0–1)	0	0.00 (0–0)	0	0.00 (0–0)	1	0.00 (0–1)	n. a.

EI, eosinophilic inflammation; MI, mixed inflammation; NI, neutrophilic inflammation; HC, healthy cats; n.a., not applicable.

Two cats have had clinical signs of FLAD for <1 year at the time of presentation; therefore, seasonality could be assessed only in the remaining 22 cats. All 22 cats showed clinical signs throughout the year. While in 17 cats the severity of clinical signs did not change throughout the year, five cats had seasonal changes of severity. Of those five, two cats showed more signs in spring and three cats in summer.

Significantly more cats with FLAD (12/24, 50%) lived in a household with smoke exposure compared to HC (4/24, 17%) (*p* = 0.030). No difference was found regarding this factor when cats with EI, MI and NI were compared (*p* = 0.641).

In the FLAD group, no difference could be detected between cats eating mainly dry food and those eating moist food in reactions to SM (*Tyrophagus putrescentiae, Acarus siro, Lepidoglyphus destructor*) on IDT (*p* = 0.629). No calculation was done on SAT as only one cat showed a reaction to one of the mite allergens.

There was no difference in median positive reactions per cat for “outdoor-allergens” such as weed, grass, and tree pollen in the FLAD group between cats living exclusively indoors (median: 0.00 on SAT, median: 4.00 on IDT) and cats with outdoor access (median: 0.00 on SAT, median: 3.5 on IDT) on SAT (*p* = 0.630) or IDT (*p* = 0.252), respectively.

## 4 Discussion

Experimentally induced FA has been shown to elicit positive allergy test results (16, 20). In addition, application of AIT resulted in resolution of induced clinical signs and airway inflammation, strongly supporting the hypothesis that aeroallergens can trigger an allergic reaction causing clinical signs of FLAD (17, 26). To date, only few studies have been investigating the role of environmental allergens in cats with naturally occurring inflammatory airway disease.

In the present study HC showed more positive reactions per cat than cats with FLAD on SAT, questioning the diagnostic accuracy of allergy testing in cats with FLAD. In contrast to this finding, two other studies detected significantly more positive reactions in cats with naturally occurring FLAD compared to HC (9, 28). One of these studies that compared results from SAT and IDT in ten HC and ten cats with not further classified FLAD detected more positive reactions in both tests in affected cats compared to HC (28). Similarly, the second study found that 15 cats with EI and MI had more positive allergen reactions on SAT compared to nine HC (9). However, both studies included only small patient groups which might have influenced the statistical power. Another explanation for different findings in the present study could be the measurement methods used for SAT. In the present study, a monoclonal antibody methodology was used where antibodies may falsely bind irrelevant IgG antibodies (35). For higher specificity, the alpha chain of the high-affinity mast cell receptor for IgE (FcεR1α) is recommended as binding site for antibodies used for testing (18, 35). However, only Moriello and coworkers (28) used the FcεR1α-receptor measurement in their study. Buller and coworkers (9) performed SAT using polyclonal antibody measurement which, compared to the FcεR1α-receptor measurement, is more likely to cause false positive results (35). Another possible explanation could be a differing deworming status for cats in different studies. Referring to a study investigating pruritic cats, non-dewormed cats showed significantly more positive reactions for allergen-specific IgE than dewormed cats. The authors assumed that the presence of intestinal parasites could lead to production of IgE cross-reacting against environmental allergens (36). Deworming status was unknown for most cats in the present study as well as in the study performed by Moriello et al. (28), making it impossible to compare allergy test results regarding that point. In the study performed by Buller and coworkers on the other hand, all cats were regularly dewormed. As most cats with FLAD and HC in the present study were indoor only cats, it is unlikely that infestation with parasites could have explained the higher number of positive reactions on SAT in HC compared to cats with FLAD. Moreover, a functional heterogeneity due to different glycosylation of allergen-specific IgE could be the cause for positive reactions in healthy animals leading to the assumption that “pathogenic” and “non-pathogenic” IgE should be considered (37, 38). However, the results of the present study are consistent with findings in cats with feline atopic skin syndrome (FASS), in which no difference in allergen-specific IgE could be detected in HC compared to cats with FASS (37–40). These findings emphasize that, considering AIT as a potential treatment of FA, SAT should only be used as a guidance for allergen selection and results should always be evaluated in context with clinical signs and environmental factors, as previously recommended for treatment of FASS (33, 41, 42).

Due to ethical reasons, we could not perform IDT in HC, therefore it is not known whether more HC than cats with FLAD would have shown positive reactions on IDT as it was the case on SAT. In one study on cats with FASS it could be seen that more cats with FASS showed positive reactions on IDT than HC (43). This is in concordance with the study from Moriello et al. (28). This leads to the assumption that in our study more cats with FLAD than HC would have shown positive reactions on IDT. These findings could be explained with the high sensitivity of the test. Furthermore, IDT rather faces problems of subtle wheal formation, making it hard to read compared to dogs (44), meaning that rather some relevant allergen reactions might be missed than false positive reactions generated in HC.

Significantly more positive reactions per cat were seen on IDT compared to SAT in the present study. Previously, in a study evaluating allergy test results in cats with induced FA sensitized with bermuda grass allergen or HDMA, IDT showed higher sensitivity compared to SAT (18). In the same study, good agreement was found between allergens tested in both tests; however, because of the experimental study design allergens were known in that investigation limiting the ability to compare the results with patients suffering from naturally acquired disease.

When positive results of allergens evaluated in both tests were compared with cohen's kappa, only *Alternaria alternata* and English Plantain showed “moderate agreement.” This is consistent with results from studies on FASS (37, 45). In one study eight cats diagnosed with FASS had positive IDT results but tested negative on SAT (45). In another study comparing allergy test results for HDMA in cats with FASS, only weak correlation was found between results in both tests (37). One reason for the discrepancies between both tests could be different detection methods for allergen-specific IgE. While free circulating IgE is detected in serological assays, IDT reveals IgE bound to the FcεR1α-receptor on dermal mast cells (40). In contrast to IgE circulating in blood, dermal mast cell-bound IgE shows a significantly longer half-life, resulting in positive reactions lasting longer on IDT compared to a shorter period of detection in SAT (46). Furthermore, it can be assumed that irregular allergen exposure could also lead to the absence of circulating IgE. This might explain a higher sensitivity of IDT compared to SAT, therefore potentially “missing” some relevant allergens when using SAT.

Ten cats with FLAD in the present study showed reactions to SM on IDT, but no association with dry food diet could be found in these patients. A previous study also investigated IDT results in cats with naturally occurring FLAD in the context with environmental factors, using information gained by an owner questionnaire. Three of the six cats that showed reactions to SM reached remission of clinical signs when dry food was changed to moist food diet (47). This was also mentioned in an abstract reporting that removal of dry food led to clinical remission in 2/5 cats with naturally occurring FLAD and positive reactions to SM (48). However, in dry dog food, contamination with SM is usually very low or undetectable and therefore with the correct storage conditions quite unlikely (49). It can be assumed that these findings account for dry cat food as well, although no specific studies on SM contamination of dry cat food exist so far. In the present study, however, cats that showed reactions to SM were not changed to moist food to assess a potential clinical improvement related to a dietary change.

No difference could be detected when indoor cats and cats with outdoor access were compared in the present study regarding positive reactions per cat on environmental allergens in both tests. This finding stands in contrast to results from a study on pruritic cats, suggesting that outdoor cats are consistently challenged by environmental allergens, endo-, and ectoparasites and therefore tend to have more positive reactions on SAT. In that study, outdoor cats showed more positive reactions to food and environmental allergens compared to indoor cats (36).

Seasonality has scarcely been assessed in cats with naturally occurring FLAD. One study mentioned more intense signs and episodes of respiratory distress in five cats during summer, in two during autumn and in two during winter. The remaining 16 cats did not show any seasonal changes (12). In the present investigation, only five cats revealed stronger clinical signs during spring and summer. It can be assumed that seasonality is not common in cats with FLAD.

On SAT the most common positive reactions in cats with FLAD were seen for American house dust mite (*Dermatophagoides farinae*). This is not surprising, as HDMA has been shown to be one of the most prevalent allergens cats are sensitized to in several studies on FASS (37, 45). In addition, cats with FA were commonly sensitized to this allergen on SAT in previous studies (9, 29). One study compared SAT results in cats with FA to those in HC and interestingly, reactions to HDMA were only found in cats with FLAD (9). In contrast to that, in the present study house dust mite was shown to be one of the most common reactions on SAT in HC as well as in affected cats. However, in the previous study only nine cats served as a control group and just one of those showed positive reactions on SAT, which potentially underestimates the most common allergen reactions in HC (9).

While in the present study house dust mite was not one of the most common allergen reactions on IDT in cats with FLAD, in two other studies using IDT for allergen testing, reactions to American house dust mite in 8/15 cats showing positive reactions (47) and 4/9 cats showing positive reactions to this allergen (48), represented the most common positive skin reactions in cats with FLAD (47, 48).

To date, no studies have been investigating allergy test results in the context of different types of airway inflammation. In the present study, for most allergen groups no significant differences could be detected between median positive reactions per cat comparing EI, MI, NI and HC. However, cats with EI showed significantly more positive allergen reactions compared to those with MI for tree pollen and insects, and cats with NI had significantly more positive reactions to insects compared to cats with MI. These findings stand in contrast to the assumption that EI is a consequence of an allergic reaction. Similar to human asthma, FLAD might also have a non-allergic non-IgE-mediated etiology in cats, therefore overestimating the role of allergens in cats with airway disease and potentially explaining the overall low rate of positive reactions in SAT in cats with FLAD in the present study.

In general, categorization into different inflammatory types based on BALF cytology alone remains questionable, since definitions and cytology cut-off values for the different groups vary in many studies (6, 9, 10, 12). Therefore, it would have had an impact on the results of this study, if a different classification scheme had been chosen, emphasizing the problem of non-existing standardized cutoff-values. In addition to that, it has been shown that BALF samples from different lung segments of cats revealed different predominance of inflammatory cells in BALF cytology (30), raising the question whether FA and CB represent different conditions with different etiologies at all. It seems not unlikely that NI can develop as a consequence of an allergic reaction to environmental allergens as well since in one study NI could be induced experimentally by allergen exposure in research cats (2). Likewise, horses with severe equine asthma commonly express a neutrophilic inflammatory response associated with exposure to environmental allergens (50, 51). In humans, allergen-induced severe asthma is commonly associated with a neutrophilic inflammatory pattern as well (52).

CB in cats as a non-septic inflammatory condition of the lower airways with involvement of non-degenerated neutrophils is thought to arise secondarily to insults of the airways induced by multiple possible irritants, one of them being cigarette smoke in humans (1, 53). It could be shown that cigarette smoke can generate various lesions in the epithelial cells of human airways (54). In the present study, significantly more cats with FLAD lived in a smoker-household compared to HC. Nonetheless, cats with NI were not overrepresented in the group of cats from smoker households. However, to investigate the potential role of smoke exposure in the pathogenesis of FLAD, a larger sample size of cats with FLAD and HC would be needed to evaluate this factor.

There are several limitations of this study. One of them was the evaluation of the cats' environment dependent on their owners' subjectivity in the questionnaire. In addition, the impact of seasonality on clinical signs was also assessed by the cat owners only. Allergens tested in this study might not have reflected all relevant allergens present in the cats' environment, but to date, data on relevant allergens inducing sensitization in cats are limited (55). Therefore, some relevant allergens including food allergens might not have been tested in the study population. To evaluate potential differences in allergen profiles between the three types of airway inflammation, studies with distinctively larger groups are warranted. Since most cats included in this study showed perennial signs and were therefore more likely sensitized against perennial allergens such as HDMA, it would be interesting to collect dust samples in the cats' environment for investigation of mite exposure, since HDMA seems to be a relevant allergen in cats with FLAD (56).

## 5 Conclusions and relevance

Cats with FLAD do not show more positive reactions to environmental allergens on SAT than HC. In addition, correlation for most allergens is weak between SAT and IDT. Therefore, positive reactions to environmental allergens primarily demonstrate exposure to allergens but need to be interpreted with some caution in patients with FLAD. For selection of allergens for AIT, interpretation of results should always be performed in the context of clinical signs and allergen exposure for the individual cat.

## Data availability statement

The original contributions presented in the study are included in the article/Supplementary material, further inquiries can be directed to the corresponding author.

## Ethics statement

The animal studies were approved by the Ethics Committee of the Centre for Clinical Veterinary Medicine of the Ludwig Maximilian University of Munich, Germany (No. 239-16-11-2020). The studies were conducted in accordance with the local legislation and institutional requirements. Written informed consent was obtained from the owners for the participation of their animals in this study.

## Author contributions

BH: Writing—original draft. RM: Conceptualization, Data curation, Project administration, Supervision, Writing—review & editing. JG: Data curation, Writing—review & editing. TW: Conceptualization, Writing—review & editing. TB: Conceptualization, Writing—review & editing. JP: Conceptualization, Methodology, Writing—review & editing. BS: Conceptualization, Data curation, Formal analysis, Investigation, Methodology, Project administration, Supervision, Validation, Writing—review & editing.
