# Supplementary material for: Reactions to environmental allergens in cats with feline lower airway disease
Source: Front Vet Sci. 2023 Dec 7;10:1267496. doi: 10.3389/fvets.2023.1267496 (PMC10734688; doi:10.3389/fvets.2023.1267496)
Supplement: Supplementary file 1 [file Table_1.docx]

| EI (Cat No.) | Number of positive reactions (IDT) | Number of positive reactions (SAT) | MI (Cat No.) | Number of positive reactions (IDT) | Number of positive reactions (SAT) | NI (Cat No.) | Number of positive reactions (IDT) | Number of positive reactions (SAT) |
| --- | --- | --- | --- | --- | --- | --- | --- | --- |
| 1 | 11 | 5 | 9 | 9 | 0 | 19 | 10 | 0 |
| 2 | 9 | 7 | 10 | 5 | 1 | 20 | 7 | 0 |
| 3 | 14 | n.a. | 11 | 0 | 4 | 21 | 5 | 0 |
| 4 | 8 | 9 | 12 | 5 | n.a. | 22 | 4 | n.a. |
| 5 | 5 | 0 | 13 | 12 | 0 | 23 | 5 | 10 |
| 6 | 5 | 7 | 14 | 1 | 0 | 24 | 5 | 1 |
| 7 | 12 | 0 | 15 | 1 | 4 |  |  |  |
| 8 | 6 | 0 | 16 | 5 | 0 |  |  |  |
|  |  |  | 17 | 7 | 1 |  |  |  |
|  |  |  | 18 | 0 | 6 |  |  |  |

**Number of positive reactions per cat (supplementary material)**

EI= Eosinophilic inflammation, NI= Neutrophilic inflammation, MI= Mixed inflammation

n.a.: not applicable (serum sample not available)
