# Supplementary material for: Reactions to environmental allergens in cats with feline lower airway disease
Source: Front Vet Sci. 2023 Dec 7;10:1267496. doi: 10.3389/fvets.2023.1267496 (PMC10734688; doi:10.3389/fvets.2023.1267496)
Supplement: Supplementary file 2 [file Data_Sheet_1.zip › Questionairre for cats showing respiritory problems.DOCX]

Label

Date:________________

# Questionnaire on cats with inflammatory bronchial diseases

**How long has your cat been showing respiratory problems?**

⃝ _____Weeks ⃝ _____Months ⃝ ______Years

**How was your cat living at the time the respiratory problems started?**

⃝ Outdoor cat ⃝ Indoor cat ⃝ Balcony/terrace only

**What kind of environment were you living in at that time?**

⃝ Big City ⃝ Small Town ⃝ Village

**Are the symptoms:**  ⃝ Seasonal -> ⃝ Spring ⃝ Summer ⃝ Autumn ⃝ Winter

⃝ First seasonal, now non-seasonal ⃝ non-seasonal (all year round)

**Where does your cat come from:**  ⃝ Farm ⃝Animal welfare/animal shelter ⃝ Private offspring

⃝ Cat breeder

**Is your cat exposed to tobacco smoke in your home?**  ⃝ Yes ⃝ No ⃝ Occasionally

**Do you or other family members smoke outside the house/apartment?**  ⃝ Yes ⃝ No

⃝ Occasionally

**What diet has your cat been fed until the onset of symptoms?**

⃝ Commercial dry food

⃝ Commercial moist food

⃝ Moist and dry food

⃝ Home-cooked

⃝ BARF

⃝ Other

**Is there an open fireplace/stove in the house?** ⃝ Yes ⃝ No

**What cat litter was used at the time of symptom onset?**

___________________________

**At the time of the first symptoms, did you have other animals in your household or did your cat have regular contact with other animals?** ⃝ No

⃝ Yes, namely: ⃝ Dog

⃝ Cat

⃝ Small mammals (rabbits, etc.)

⃝ Horse

⃝ Bird

⃝ Other ___

**How would you rate the intensity of dust exposure in your environment?**  ⃝ None

(Assessment in regard of construction sites, particulate matter, factories, etc.)

⃝ Low

⃝ Moderate

⃝ High

**What kind of floors do you mainly have in your apartment/house?**  ⃝ Carpet ⃝ Parquet

⃝ Linoleum ⃝ Tiles

**Do you use fragrance sprays in your home?**  ⃝ No ⃝ Yes, namely:___

**Do you have a humidifier?**  ⃝ Yes ⃝ No

**What types of trees are common in your area?**⃝ Birch ⃝ Beech ⃝ Oak

⃝ Poplar ⃝ Larch ⃝ Maple

⃝ Lime ⃝ Alder ⃝ Willow

⃝ Walnut (hazel) ⃝others, namely: ___________

**Have you had mold infestation in your home?**  ⃝ Yes ⃝ no

**Do you have cat grass in your home?**  ⃝ Yes ⃝ No

**Do you live in an old apartment/house?** ⃝Yes ⃝ No

**Do you have goose feathers/down at home? (bed linen, winter down jacket)?**

⃝Yes ⃝ No

**Do you have something made of sheep's wool at home?**  ⃝ Yes ⃝ No

**Does your pet have skin problems?**  ⃝ No ⃝ Yes, since when? ____

(If you have ticked "Yes", please fill in the following pages, if you have ticked "No" the questionnaire ends here)

_____________ ______________________

Location, Date Owner's Signature

**Did skin problems appear first or respiratory problems first?**  ⃝ Skin problems first

⃝ Respiratory problems first

**Does your pet also react with gastrointestinal signs to certain diets?**

⃝ Diarrhea

⃝ Vomiting

⃝ Not applicable

**Have you ever consulted a veterinarian for the skin?**  ⃝ Yes ⃝ No

If so, when? _______

What diagnosis has been made in relation to the skin: ___________

**Does you cat have any other diseases?**  ⃝ No ⃝ Yes, namely:____________

**What kind of skin problems does your cat show?**

⃝ Itching ⃝ Redness of the skin ⃝ Dandruff ⃝ Crusts

⃝ Nodules ⃝ Hair loss ⃝ Ocular discharge ⃝ Blackening of the skin

⃝ Dull coat ⃝ Oily skin

**Which parts of the body are affected by skin problems (multiple answers possible)?**

⃝ Neck/Chest ⃝ Back ⃝ Head ⃝ Ears

⃝ Armpits ⃝ Abdomen ⃝ Groin ⃝ Sides ⃝ Tail (base) ⃝ Paws

**How severe is the itching?**

**No itching Extreme itching**

**In which months do the symptoms appear and how severe are they?**

All year round ⃝, or ⃝ :

|  |  |  |  |  |  |  |  |  |  |  | *Severe*  *itching* |
| --- | --- | --- | --- | --- | --- | --- | --- | --- | --- | --- | --- |
|  |  |  |  |  |  |  |  |  |  |  | *Moderate*  *itching* |
|  |  |  |  |  |  |  |  |  |  |  | *Lighter*  *itching* |

**Does your pet have a known food allergy? ⃝** No ⃝ Yes, against_______________

**Does your pet need medication at the moment?** ⃝ No ⃝ Yes, namely:_________

**When was the last time your pet was given the following medications?**

⃝ Cortisone: _________

⃝ Apoquel: ____

⃝ Antihistamines: _________

⃝ Ear medication (provide name): __________

**Has an allergy test already been performed?**⃝ No ⃝ Yes, namely: ⃝ Skin test ⃝ Serum allergy test

Results:________________________________________________________________________________________________________________________________________________________________________________________________________________________________________________________________________________________________________________________________________

Has desensitization been performed on your pet? ⃝ Yes ⃝ No

_____________ ______________________

Location,Date Owner's Signature
