# Supplementary material for: Reactions to environmental allergens in cats with feline lower airway disease
Source: Front Vet Sci. 2023 Dec 7;10:1267496. doi: 10.3389/fvets.2023.1267496 (PMC10734688; doi:10.3389/fvets.2023.1267496)
Supplement: Supplementary file 2 [file Data_Sheet_1.zip › Questionairre for healthy cats.DOCX]

Label

Datum:________________

# Questionnaire on cats with inflammatory bronchial diseases

**Is your cat showing respiratory problems?**

⃝ Yes ⃝ No

**How does your cat live?**

⃝ Outdoor cat ⃝ Indoor cat ⃝ Balcony/terrace only

**What kind of environment?**

⃝ Big City ⃝ Small Town ⃝ Village

**Where does your cat come from:**  ⃝ Farm ⃝ Animal welfare/animal shelter ⃝ Private offspring

⃝ Cat breeder

**Is your cat exposed to tobacco smoke in your home?**

⃝ Yes ⃝ No ⃝ Occasionally

**Do you or other family members smoke outside the house/apartment?**

⃝ Yes ⃝ No

⃝ Occasionally

**What** **diet is fed?**

⃝ Commercial dry food

⃝ Commercial moist food

⃝ Moist and dry food

⃝ Home-cooked

⃝ BARF

⃝ Other

**Is there an open fireplace/stove in the house?** ⃝ Yes ⃝ No

**What cat litter is used?** ___________________________

**Do you have other animals in the household or does your cat have regular contact with other animals?** ⃝ No

⃝ Yes, namely:

⃝ Dog

⃝ Cat

⃝ Small mammals (rabbits, etc.)

⃝ Horse

⃝ Bird

⃝ Other ___

**How would you rate the intensity of dust exposure in your environment?**  ⃝ None

(Assessment in regard of construction sites, particulate matter, factories, etc.)

⃝ Low

⃝ Moderate

⃝ High

**What kind of floors do you mainly have in your apartment/house?**  ⃝ Carpet ⃝ Parquet

⃝ Linoleum ⃝ Tiles

**Do you use fragrance sprays in your home?**  ⃝ No ⃝ Yes, namely:___

**Do you have a humidifier?**  ⃝ Yes ⃝ No

**What types of trees are common in your area?** ⃝ Birch ⃝ Beech ⃝ Oak

⃝ Poplar ⃝ Larch ⃝ Maple

⃝ Lime ⃝ Alder ⃝ Willow

⃝ Walnut (hazel) ⃝ others, namely_____________

**Have you had mould infestation in your home?**  ⃝ Yes ⃝ No

**Do you have cat grass in your home?**  ⃝ Yes ⃝ No

**Do you live in an old apartment/house?** ⃝Yes ⃝ No

**Do you have goose feathers/down at home? (bed linen, winter down jacket)?**

⃝Yes ⃝ No

**Do you have something made of sheep's wool at home?**  ⃝ Yes ⃝ No

**Does your pet show skin problems?**  ⃝ No ⃝ Yes

_____________ ______________________

Location, Date Owner's Signature
